# Müllerian Serous Cystadenoma of the Scrotum Following Orchiopexy

**DOI:** 10.1155/2009/610453

**Published:** 2009-03-30

**Authors:** Sebastian C. J. van der Putte, Johan Toonstra, Daisy M. D. S. Sie-Go

**Affiliations:** ^1^Department of Pathology, University Medical Centre Utrecht, Utrecht, The Netherlands; ^2^Department of Dermatology, University Medical Centre Utrecht, Utrecht, The Netherlands

## Abstract

A 24-year-old man presented himself with a nodular lesion of about 1 cm diameter at the site of a previous orchiopexy associated with surgery for cryptorchism. Histopathology revealed the lesion to be adenomatous and confined to the scrotum. Histological and immunohistological features were not consistent neither with median raphe cysts or cutaneous adenomas nor with the intrascrotal adenomas of the rete testis, epididymis, nor with (malignant) mesotheliomas. However, the lesion did compare well with serous (papillary) cystadenomas of the testis or paratestis. These adenomas are thought to originate in remnants of the Müllerian system or of peritoneal lining altered by Müllerian metaplasia. This implies that the scrotal adenoma may have developed from an implant of such elements during orchiopexy 14 years ago. Complete excision of the lesion appears to be an adequate therapy.

## 1. Introduction

Tumors and tumor-like lesions in the scrotum comprise a wide spectrum of conditions related to the scrotum and to the testis and paratestis [1, 2]. These two categories of “scrotal” and “intrascrotal” lesions are strictly separated by the anatomical barrier of the tunica vaginalis.

In 2004, Hara et al. [3] described an adenomatous tumor which appeared to have developed at the site of an orchiopexy performed two weeks earlier and involved the scrotum as well as the testis. They diagnosed the lesion as a median raphe cyst with features of a borderline serous cystadenoma. In 2007, Dehner et al. [4] reported a patient with a similar condition who had been operated on seven years earlier and classified the lesion as a median raphe cyst. We present a third case which offered an extra diagnostic challenge by absence of involvement of the testis or paratestis. On the basis of histopathological criteria, we dismiss the diagnosis of median raphe cyst and classify the disorder as a serous cystadenoma originating in a Müllerian implant in the scrotum.

## 2. Case Presentation

A 24-year-old male noticed a circumscribed swelling in the skin of his left scrotum. Physical examination showed a 1.5 cm nodus at the site of an orchiopexy performed for cryptorchism 14 years earlier. It was excised under local anesthesia under the suspected diagnosis of pilar cyst. No connection with intrascrotal structures was noticed.

**Figure 1:**
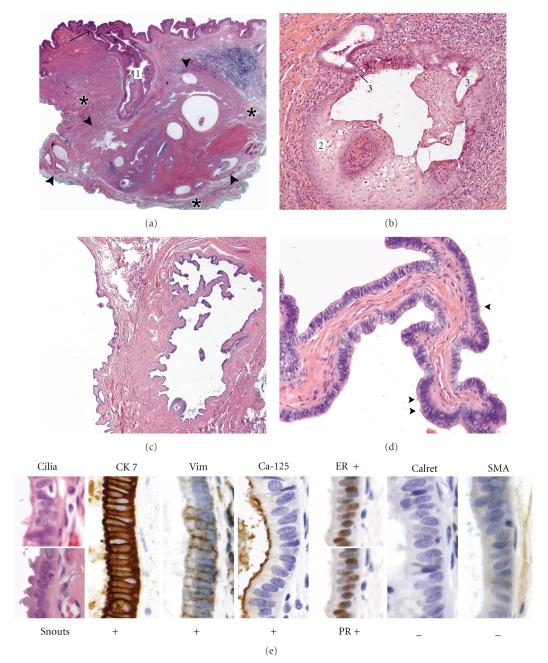
Histological and immunohistochemical features of the scrotal serous cystadenoma at the side of an orchiopexy 14 years earlier. (a) Low magnification shows a tubulocystic tumor (between large arrowheads) surrounded by connective tissue and embedded in the dartos fascia (asterisks). Note its connection to the epidermis by a sinus (1) lined by cornifying stratified squamous epithelium and opening at the surface (arrow). (b) Detail of metaplastic noncornifying stratified squamous epithelium (2) passing into a single-layered columnar epithelium of the tubulocystic system (3) in the deepest part of the sinus. (c) Cystic papillary component. (d) Detail of a fibrous papilla covered by characteristic tall columnar epithelium with ciliated cells (small arrowheads). (e) Cytological and immunohistochemical characteristics discriminating the tumor from other scrotal and intrascrotal lesions. Vim: Vimentin; ER: Estrogen receptor; PR: Progesterone receptor; Calret: Calretinin; SMA: Smooth muscle actin.

Macroscopy showed an ellipsoid 2.5 × 1.5 × 1 cm specimen with a firm 1 cm tumor. Microscopic sections revealed an irregular tubulocystic epithelial proliferation surrounded by a mantle of connective tissue and embedded in the smooth musculature of the dartos fascia (Figure 1(a)). The tumor was completely removed with a very narrow free margin. The tubulocystic element spread outward from a central sinus that opened externally at the surface of the skin and was lined by a cornifying stratified squamous epithelium (Figure 1(a)). Metaplastic noncornifying stratified squamous epithelium in the deepest part of the sinus passed into columnar to cuboidal epithelium in the tubulocystic component of the tumor (Figure 1(b)). In addition to the tubulocystic element, slit-like and papillary configurations were also observed (Figures 1(c), 1(d)). Variable numbers of cells showed prominent apical snouts or cilia (Figures 1(d), 1(e)). The epithelium was bland, mitotic figures were rare, and microinvasion was absent. Psammona bodies were not observed.

A specially selected differential diagnostic panel of immunohistochemical tests demonstrated reactivity for cytokeratin (CK) 7 (Biogenex, San Ramon, USA), vimentin (DAKO, Glastrup, Denmark), Ca-25 (Novocastra, Newcastle, UK), estrogen receptor (ER), progesterone receptor (PR) (DAKO), nonreactivity for CK 20 (DAKO), calretinin (Klinipath, Duiven, The Netherlands), and alpha-smooth muscle actin (SMA) (Sigma, St Louis, USA). This profile was inconsistent with scrotal median raphe cysts (nonreactive for vimentin) (three cases tested) and cutaneous papillary hidradenomas (reactive for SMA in myoepithelium) (three cases tested). With respect to the possibility of a derivation from paratesticular structures (three cases tested), the immunoprofile of the tumor did not match those of the rete testis and mesothelium (nonreactive for Ca-125, ER, and PR, but reactive for calretinin) or epididymis (nonreactive for Ca-125 and variably reactive for vimentin, ER, and PR). However, in concordance with cytological details, the profile of the tumor is similar to that of serous (papillary) cystadenoma of the ovary (three cases tested). The MIB1 cell proliferation index was 1.5%.

## 3. Discussion

The present case appears to be almost identical in clinical and histopathological aspects to those described by Hara et al. [3] and Dehner et al. [4]. It differed in the longer interval between the orchiopexy and the manifestation of the anomaly and its confinement to the scrotum. Their argumentation, in favor of a classification of the lesion as a median raphe cyst though, was not convincing.

Median raphe cysts are localized in the midline between the preanal perineum and the external urinary meatus and typically show a urethra type of epithelium which is pseudostratified columnar epithelium with occasional mucus production or cilia and often undergoes squamous metaplasia [5–8] (own unpublished observations in 19 cases). Cysts near the meatus predominate and can be explained by a derivation from ectopic urethral glands [9]. Cysts (and canals) related to the rest of the raphe, which is at some distance from the urethra and its glands, are thought to have grown from the embryonic urogenital sinus epithelium which had remained in the raphe after it had been formed by fusion of the urethral folds during the formation of the phallic urethra [5, 7, 8]. This theory has to be dismissed as it has recently been established that such fusion does not take place [10]. However, the idea that raphe cysts derive from urogenital sinus epithelium can be upheld as the same study also revealed that this epithelium temporarily lines the cloacal groove which derives from the middle segment of the cloaca between the urogenital and anal compartments of the cloaca and at the surface of the perineum elongates into the male raphe later. Both the urethra type of epithelium and the direct relationship with the raphe are lacking in the lesions described by Hara et al. [3] and Dehner et al. [4] and also in our case.

The possibility that the lesion had developed from cutaneous glands had to be discarded as well. Such adenomas characteristically show a basal layer of smooth muscle actin-positive myoepithelium [11] which was missing in our case.

The absence of a primarily scrotal alternative diagnosis, the localization at the site of the orchiopexy, and the involvement of the paratestis as reported by Hara et al. [3] and Dehner et al. [4] suggest a paratesticular derivation. Among the adenomatous tumors in this category, the adenomatoid tumor [1, 12, 13] and papillary cystadenoma of the epididymis [1, 12–14] have a distinctly different morphology and a different immunoprofile. The cystadenoma of the rete testis and (malignant) mesothelioma may have a certain morphological resemblance but differ in immunoprofile especially by showing reactivity for calretinin [12].

The histological and immunohistochemical features of the lesion were indeed well in accordance with those of the serous (papillary) cystadenomas of the paratestis (and ovary) [12, 13, 15] as discovered by Hara et al. [3]. These serous cystadenomas are rare tumors which become manifest as a painless swelling in mostly middle-aged men and appear to be similar to their ovarian counterparts. Mild atypia in combination with stratification may lead to the suspicion of borderline malignancy but prognosis after complete removal was excellent [13]. Mitotic activity was low.

These cystadenomas are thought to originate in remnants of the Müllerian (paramesonephric) system and/or by Müllerian metaplasia of the peritoneal lining of the tunica vaginalis [1, 12]. In the present specific situation of an isolated scrotal localization, this would mean that the lesion is the result of implantation of Müllerian tissue in the incision for the orchiopexy. It is not clear if the external opening in our patient and the drainage in the case of Dehner et al. [4] are a primary or secondary event.

The precise nature of the tumor is still obscure. The presumed development of the lesion of Hara et al. [3] within two weeks after the operation appears highly unusual for an adenoma. The report of three cases of this adenoma in relation with orchiopexy as compared with the report of less than 40 cases of testicular and paratesticular cystadenoma in the general male population [12] is remarkable and suggests some sort of special stimulus on the ectopic tissue. The growth of the tumor over a twenty-one-month period as observed by Hara et al. [3] demonstrates a progressive character. Their grading of the lesion as a borderline malignancy may be disputed because mild atypia in some epithelial cells as the only indicator falls far short of criteria currently applied to the ovarian counterpart [16]. The disease-free follow-up periods of at least 40 months [3] and 14 months (our patient) suggest that, like the testicular or paratesticular cystadenoma, complete excision may be adequate but it is evident that more data are needed.

## 4. Conclusions

Orchiopexy may be complicated by an adenomatous tumor morphologically identical to the Müllerian serous (papillary) cystadenoma of the paratestis. Confinement of the present lesion to the scrotum suggests implantation of Müllerian elements during the operation. The previous designation “median raphe cyst mimicking a serous borderline tumor” is incorrect. The still-limited data available suggest that complete excision may be an adequate therapy.
